# Time-locked acute alpha-frequency stimulation of subthalamic nuclei during the evaluation of emotional stimuli and its effect on power modulation

**DOI:** 10.3389/fnhum.2023.1181635

**Published:** 2023-07-27

**Authors:** Naeem Muhammad, Saurabh Sonkusare, Qiong Ding, Linbin Wang, Alekhya Mandali, Yi Jie Zhao, Bomin Sun, Dianyou Li, Valerie Voon

**Affiliations:** ^1^Department of Neurosurgery, Centre for Functional Neurosurgery, Ruijin Hospital, Shanghai Jiao Tong University School of Medicine, Shanghai, China; ^2^Department of Psychiatry, University of Cambridge, Cambridge, United Kingdom; ^3^Institute of Science and Technology for Brain-Inspired Intelligence, Fudan University, Shanghai, China; ^4^Key Laboratory of Computational Neuroscience and Brain-Inspired Intelligence, Fudan University, Shanghai, China

**Keywords:** deep brain stimulation (DBS), acute stimulation, alpha frequency, emotion, event related (de)/synchronization

## Abstract

**Introduction:**

Deep brain stimulation (DBS) studies in Parkinson's Disease (PD) targeting the subthalamic nucleus (STN) have characterized its spectral properties across cognitive processes. In emotional evaluation tasks, specific alpha frequency (8–12 Hz) event-related de-synchronization (ERD) (reduced power) has been demonstrated. The time-locked stimulation of STN relative to stimuli onset has shown subjective positive valence shifts with 10 Hz but not with 130 Hz. However, neurophysiological effects of stimulation on power modulation have not been investigated. We aim to investigate effects of acute stimulation of the right STN on concurrent power modulation in the contralateral STN and frontal scalp EEG. From our previous study, we had a strong *a priori* hypothesis that negative imagery without stimulation would be associated with alpha ERD; negative imagery with 130 Hz stimulation would be also associated with alpha ERD given the lack of its effect on subjective valence ratings; negative imagery with 10 Hz stimulation was to be associated with enhanced alpha power given the shift in behavioral valence ratings.

**Methods:**

Twenty-four subjects with STN DBS underwent emotional picture-viewing tasks comprising neutral and negative pictures. In a subset of these subjects, the negative images were associated with time-locked acute stimulation at either 10 or 130 Hz. Power of signals was estimated relative to the baseline and subjected to non-parametric statistical testing.

**Results:**

As hypothesized, in 130 Hz stimulation condition, we show a decrease in alpha power to negative vs. neutral images irrespective of stimulation. In contrast, this alpha power decrease was no longer evident in the negative 10 Hz stimulation condition consistent with a predicted increase in alpha power. Greater beta power in the 10 Hz stimulation condition along with correlations between beta power across the 10 Hz stimulation and unstimulated conditions suggest physiological and cognitive generalization effects.

**Conclusion:**

Acute alpha-specific frequency stimulation presumably was associated with a loss of this expected decrease or desynchronization in alpha power to negative images suggesting the capacity to facilitate the synchronization of alpha and enhance power. Acute time-locked stimulation has the potential to provide causal insights into the spectral frequencies and temporal dynamics of emotional processing.

## Introduction

Deep brain stimulation (DBS) is a neurosurgical procedure that involves targeted implantation of electrodes to deliver electrical pulses (Benabid et al., 2009; Lozano et al., 2019). Randomized controlled trials have shown DBS efficacy for neurological disorders such as Parkinson's disease (PD) (Benabid et al., 2009) and for psychiatric disorders such as obsessive-compulsive disorder (OCD) (Blomstedt et al., 2013; Visser-Vandewalle et al., 2022). For PD, high-frequency (e.g., 130 Hz) subthalamic nucleus (STN) stimulation is commonly used clinically to relieve motor symptoms (Benabid et al., 2009), while lower frequencies (e.g., 60 Hz) are used to target gait symptoms (Xie et al., 2017). The STN, a nucleus found in the indirect pathway of basal ganglia, receives both indirect and hyperdirect projections from the cortico-striatal-thalamo-cortical circuitry with evidence for both segregation as well as integration of motor, cognitive, and limbic substrates (Kim et al., 2015; Eisinger et al., 2019).

Complementary to the clinical benefits, DBS studies targeting the STN for PD have contributed greatly to our understanding of the STN circuits (Eisinger et al., 2019). STN recordings acquired under various task paradigms also provide knowledge on the functional role of STN in cognition and emotion. For instance, spectral dynamics of STN response in emotional evaluation tasks have demonstrated specific event-related de-synchronization (ERD) or reduced activity in the alpha frequencies (8–12 Hz) with latency nearly 0.5 s after image onset and peaking at 1–2 s (Kühn et al., 2005a; Brücke et al., 2007; Huebl et al., 2011, 2014; Buot et al., 2013). This ERD correlates with subjective emotional valence ratings (Brücke et al., 2007) and also is associated with depressive symptoms (Huebl et al., 2011). Consistent and supporting evidence about the role of alpha ERD in emotional processing also comes from other DBS studies targeting other subcortical structures such as the habenula (Sonkusare et al., 2022a). Furthermore, chronic high-frequency STN stimulation in PD enhances positive valence bias (Irmen et al., 2017), and chronic anteroventral STN stimulation in patients with obsessive-compulsive disorder enhanced the positive ratings of low-intensity negative and positive images (Voon et al., 2017). Thus, a multitude of studies implicates the involvement of STN neural oscillations, especially in the alpha band, in emotional processing.

DBS studies also offer the potential for acute stimulation which can be time locked according to the presentation of stimuli. Such refined methods of stimulation which explore the timing of stimulation delivery and its effect on subjective evaluation provide causal insights about the spectral frequencies and temporal dynamics of emotional processing as well as potential development of neuromodulation protocols. In our previous study, we specifically addressed this question showing that acute one-second alpha-specific (10 Hz) frequency stimulation of the STN time-locked to the evaluative period of affective negative imagery enhances subjective positive emotional subjective valence but not with acute clinical high-frequency (130 Hz) stimulation (Mandali et al., 2021). The effect of stimuli-locked stimulation on subjective behavioral ratings was investigated, and neurophysiological effects of stimulation were not reported. Electrodes for DBS allow either stimulation or recordings at one time but never concomitantly. In the previous study, we had stimulated right STN, while recordings from left STN but data were not analyzed. In this study, in a complementary analysis, we aimed to investigate the effect of stimulation on concurrent power modulation of the left STN neural activity. Based on our previous behavioral findings of stimulation, we had a strong *a priori* hypothesis that negative imagery without stimulation would be associated with alpha ERD and negative imagery with 130 Hz stimulation was also associated with alpha ERD given the lack of effect on behavioral subjective valence ratings. We further hypothesized that 10 Hz stimulation would facilitate or enhance alpha power during negative imagery given the shift in behavioral valence ratings.

## Materials and methods

Twenty four PD patients participated in the study: six females, age (mean ± SD) = 59.71 ± 11.84, right-handed, BDI-II = 12.54 ± 7.18. Detailed information on the recruitment criterion, patient demographics, clinical evaluation, surgical procedures, and stimulation parameters including settings and protocol are provided in the previous study (Mandali et al., 2021). All procedures utilized in the current study were approved by the ethics committee of Ruijin Hospital, Shanghai Jiao Tong University School of Medicine. Written informed consent was obtained from all participants in accordance with the Declaration of Helsinki.

### Electrode localization

**Figure 1:**
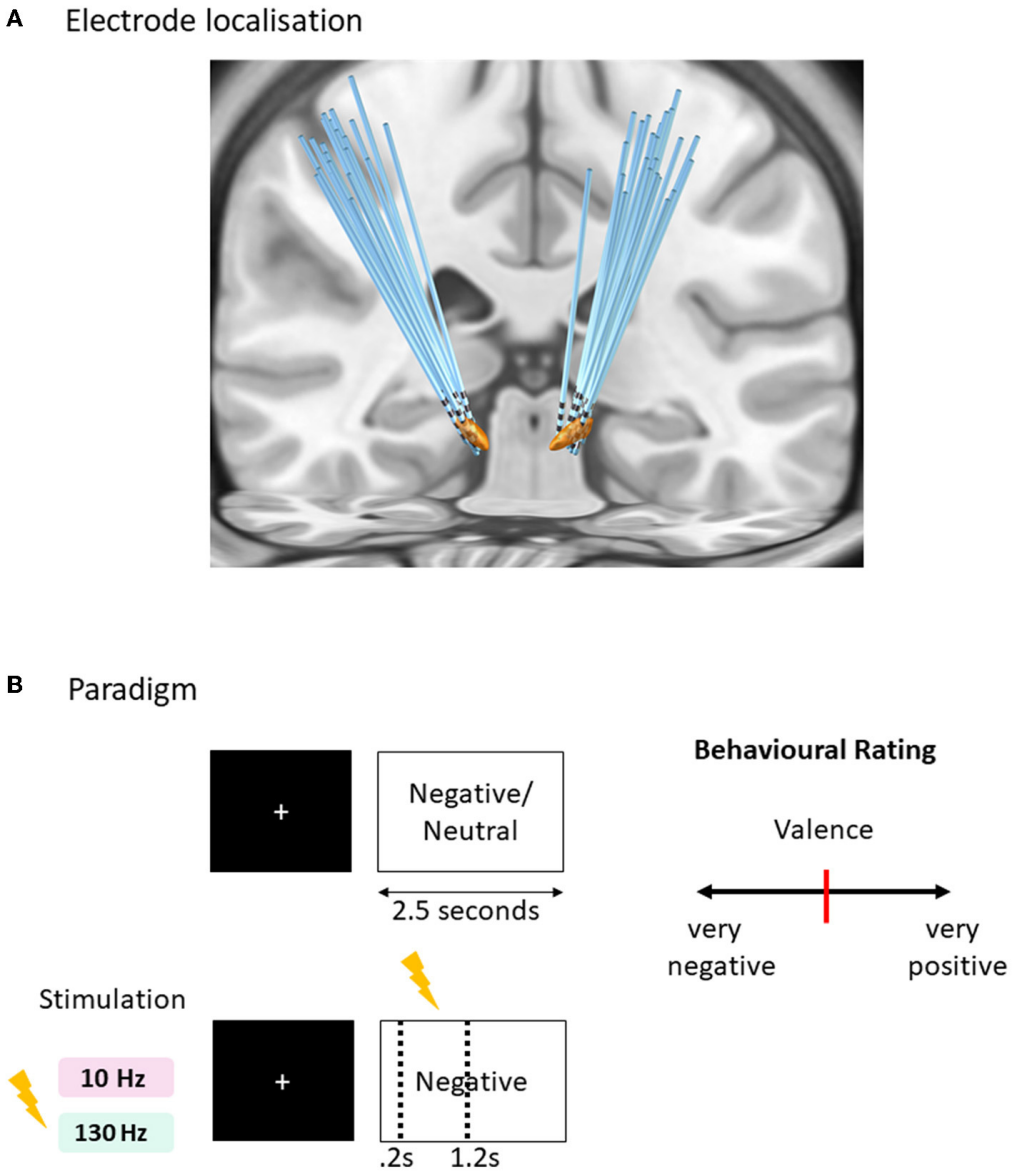
Affective task paradigm with acute time-locked stimulation. **(A)** Sequence of a typical trial in three conditions of tasks (negative and neutral without stimulation, negative with stimulation). One of the negative condition was paired with acute right subthalamic nuclei stimulation for 1 s duration after 0.2 s of the image onset lasting for 1 s, within the 2.5 s duration of the trial. Two versions of the task were created with matching valence and arousal values tested in randomized order with 130 Hz and 10 Hz stimulation frequencies. **(B)** Rating design displaying a question to score the behavioral measure of valence and arousal on a visual analog scale [ranges from 0 to 100 with initial position fixed at 50 (red bar in the center)] using the mouse. Each trial is separated with an inter-trial interval jittered between 1 and 1.5 s with a fixation cross displayed at the center of the screen.

The intended target coordinates of the subthalamic nucleus (STN) were determined by integrating post-operative computed tomography and pre-operative 3.0 Tesla magnetic resonance imaging (MRI) images within Surgiplan software (Elekta, Sweden). Quadripolar electrodes with four platinum–iridium contacts (Medtronic 3387S, Medtronic, USA; or PINS L302, PINS, China; Sceneray SR1210, China) were implanted using stereotactic navigation into the bilateral STN under general anesthesia. Participants were tested 24 h after surgery to avoid the stun effect and at least 30 min after their regular medication dose. Post-operative CT and pre-operative T1 MRI were used to reconstruct the electrode trajectories and their locations by employing the LEAD-DBS toolbox (Deiber et al., 2020) (Figure 1). Briefly, a two-stage linear registration as implemented in using Advanced Normalization Tools (ANT) (Campbell et al., 2012) was used, and the post-operative CT co-registered to pre-operative MRI and spatially normalized into MNI_ICBM_2009b_NLIN_ASYM space (Sonkusare et al., 2022a). The Pacers algorithm (Sonkusare et al., 2022b) was used to localize electrodes in MNI space.

### Paradigm

**Figure 2:**
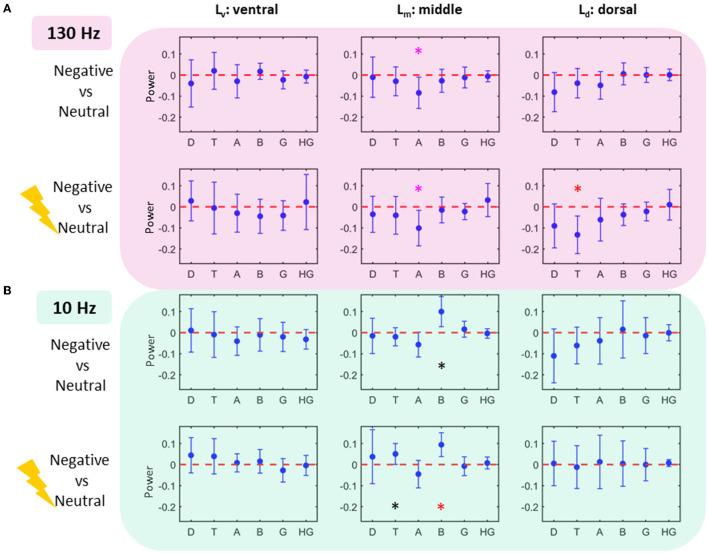
Power modulations as calculated with log-to-power ratio (LPR) **(A)** *130 Hz Stimulation Task:* Middle contact L_m_ depicts alpha power decrease for both LPR_neg_ vs. LPR_neu_ and LPR_neg130_ vs. LPR_neu_ comparisons consistent with *a priori* hypotheses (*p* < 0.05). L_d_ also shows theta decrease for LPR_neg10_ vs. LPR_neu_ comparison (*p* < 0.05, corrected). **(B)** *10 Hz Stimulation Task:* Middle contact L_m_ depicts beta increase for both LPR_neg_ vs. LPR_neu_ (*p* < 0.05; uncorrected; black star) and LPR_neg10_ vs. LPR_neu_ comparisons (*p* < 0.05; corrected; red star). Lm also shows theta increase for LPR_neg10_ vs. LPR_neu_ comparison (*p* < 0.05; uncorrected). Note that the middle contact L_m_ was not associated with alpha decrease for LPR_neg10_ vs. LPR_neu_ comparisons which is consistent with an increase in alpha power by decreasing the alpha desynchronization effect. Uncorrected significance is denoted by a black star and significance withstanding multiple comparisons corrections (*p* < 0.05) is denoted by a red star. Significance testing for *a priori* hypotheses is shown in a mauve star. Error bars denote standard deviation.

Subjects completed 90 trials of each of the emotional picture-viewing tasks (Figure 2A), utilizing pictures from the International Affective Picture System (IAPS) (Lang et al., 1997). Two tasks were run associated with either 10 or 130 Hz stimulation randomized across 2 separate days. The stimuli comprised three conditions displayed for 2.5 s randomly presented negative images associated with time-locked acute stimulation of either 10 or 130 Hz, negative images without stimulation, and neutral images without stimulation. A fixation cross was prefixed to the image presentation, the duration of which was jittered (1–1.5 s). For the stimulation-associated images, 1 s stimulation began at 0.2 s after the image, hence ending at 1.2 s after image onset. The stimulation onset was designed at delayed onset after image onset to avoid interference with early visual processing (Olofsson et al., 2008). Five images per category in each task were rated for valence and arousal (Figure 2B). Subjects were blind to the stimulation condition, and the presentation of the images with stimulation was randomized. Further details of the task paradigm are available in our previous study (Mandali et al., 2021).

The IAPS rating of stimuli within two negative conditions (with and without stimulations) for each of the two tasks was matched. IAPS ratings of stimuli in three conditions of each task are 10 Hz task negative with stimulation (valence: 2.26 ± 0.4, arousal: 5.81 ± 1.1), negative (valence: 2.46 ± 0.48, arousal: 5.85 ± 0.74), and neutral (valence: 5.03 ± 0.32, arousal: 3.1 ± 0.68) and 130 Hz task negative with stimulation (valence: 2.53 ± 0.58, arousal: 5.7 ± 0.7), negative (valence: 2.38 ± 0.45, arousal: 5.55 ± 0.77), and neutral (valence: 5.1± 0.38, arousal: 3.2 ± 0.63).

### Data acquisition

Patients were comfortably seated at a distance of 75 cm in front of a computer screen (LG, model L1954, 30 × 38 cm) with their right hand on a mouse to rate the images on visual analog scales. The experiment was coded in Psych toolbox 3 and run on MATLAB 2017 (The MathWorks, Natick, MA, USA) environment utilizing a Windows 7 desktop (Dell, Texas, USA). The local field potentials (LFP) and scalp electro-encephalogram (EEG) were recorded simultaneously using a BrainAmp MR amplifier (Brain Products, Gilching, Germany) at 500 Hz sampling rate employing a notch filter to remove 50 Hz power line interference. Intermittent stimulation was delivered *via* middle contacts of the right-STN (R1 anode and R2 cathode whereas R0 is ventral and R3 is dorsal) for 1 s at either 130 Hz or 10 Hz. The contact placement is reported in the previous publication (Mandali et al., 2021). The current pulses were delivered using a pulse generator (Scene Ray, model 1510, Suzhou, China) approved by the National Medical Products Administration, China. The precise time-based control of turning “ON” and “OFF” the stimulator was programmed within the experimental paradigm run on MATLAB, interfaced *via* a parallel port. Since the right STN was stimulated, a concurrent recording was acquired from the left STN contacts (L0, L1, L2, and L3) which were used for analyses in this study. The scalp EEG data were collected from seven frontal electrodes (Fp1, Fp2, F3, F4, F7, F8, and Fz) using the 10–20 placement system and the left mastoid as the reference channel.

### Data pre-processing

The raw data were subjected to re-referencing, filtering, trial extraction, and artifact corrections. First, the LFP data were re-referenced using a bipolar montage: subtracting data between adjacent contact pairs (e.g., L0–L1, L1–L2, and L2–L3) to extract three new LFP signals (henceforth to be referred as L_v_-ventral, L_m_-middle, and L_d_-dorsal, respectively). The aim was to mitigate volume conduction (Nunez and Cutillo, 1995). Next, a 1-Hz high-pass Butterworth filter was employed to remove potential DC offset from the extracted signals. Subsequently, trials corresponding to each condition were extracted. For analysis, the 1 s pre-stimulus baseline and the last 1.1 s of the trial period following stimulation were considered for the subsequent analysis, excluding 0.2 s after stimulation offset as a precaution for a possible contamination and rebound effect. The epoched trials were concatenated to form input signals for further processing. Finally, independent component analysis (Infomax ICA) was employed on those input signals to maximally separate similar signals and noise into separate components (Amari et al., 1995; Muhammad, 2008). Following Tukey (1977) extreme outlier samples (absolute values), three inter-quartile ranges away from the third quartile were removed from each ICs (and replaced with zeros) after pre-correcting for skewness by employing “medcouple” measure as described previously (Brys et al., 2004). The ICs were then projected back (multiplying processed ICA signals with the inverse of the un-mixing matrix) to the original signal space furnishing markedly cleaner bipolar LFP signals. Scalp EEG data were also pre-processed in a similar manner as LFP data albeit with some differences. Specifically, re-referencing involved slight variations: five bipolar EEG signals were extracted by subtracting anterior-posterior and left-right adjacent electrode pairs (F7–F3, F3–Fz, FP1–FP2, Fz–F4, and F4–F8) for further processing (henceforth referred as E1, E2, E3, E4, and E5, respectively).

### Power features

To compute power spectrum for trials of each subject, segments (baseline and three task conditions separately) of the epoched LFP and scalp EEG time series were tapered by employing a Hann window, zero padded (ten times the length of the segment) with 50% overlap for the purpose of smoothing and mitigating edge effects to estimate average spectra (*pwelch* function in Matlab). The *welch* method was employed for improved signal-to-noise ratio, especially in the presence of non-stationarities which may arise due to segmentations of time series. The average power of baseline and task conditions were estimated in *delta* (D: 2–4 Hz), *theta* (T: 4–8 Hz), *alpha* (A: 8–12 Hz), *beta* (B: 12–30 Hz), *gamma* (G: 30–60 Hz), and high *gamma* (HG: 60–200 Hz) frequency bands. For each experiment, the suppression or enhancement of power (in specific bands) of task conditions was estimated relative to the baseline as the log of the power ratio (LPR) (Pineda and Hecht, 2009).

### Non-parametric statistical analysis

The task conditions in the experiment were neutral (neu), negative (neg), negative 10 Hz stimulation (neg10), and negative 130 Hz stimulation (neg130). The LPR of each active condition was defined as LPR_neg_, LPR_neg10_, LPR_neg130_, and LPR_neu_. Statistical comparisons were employed between LPRs of negative (including stimulation) conditions vs. neutral conditions. To correct for multiple comparison corrections, the permutation method was employed to protect against false rejection of the null hypothesis: *no difference across LPR features of conditions*, at probability α = *0.05*. More specifically, data were randomly permuted across all possible pairs of LPR features extracted from conditions (within each contact and band) in the task and the experiment, and test statistics (*t-*value) was calculated. This procedure was repeated 1,000 times and the maximum absolute *t*-value was retained for each permutation. The *p*-value was estimated by the proportion that resulted in a larger test statistic than the original non-permuted observed one (Maris and Oostenveld, 2007).

## Results

### Comparison of STN power features

#### 130-Hz stimulation task

In keeping with our *a priori* hypotheses, the middle contact L_m_ showed an alpha power decrease in both LPR_neg_ vs. LPR_neu_ in the unstimulated condition and LPR_neg130_ vs. LPR_neu_ comparisons in the 130 Hz stimulated condition (*p* < 0.05) (Figure 2). The dorsal contact L_d_ showed a trend toward a theta decrease (*p* < 0.05; uncorrected) only in the case of stimulated negative condition.

#### 10 Hz stimulation task

Critically, with stimulation at 10 Hz, LPR_neg10_ vs. LPR_neu_ comparison of the middle contact no longer showed an alpha power decrease suggesting that acute 10 Hz stimulation enhanced alpha power, thus eliminating the expected alpha ERD, as predicted. The beta frequency power of the middle contact L_m_ showed LPR_neg_ vs. LPR_neu_ that showed a trend toward an increase in the unstimulated condition (*p* < 0.05; uncorrected), whereas corresponding comparison in stimulated negative condition (LPR_neg10_ vs. LPR_neu_) showed a theta increase (*p* < 0.05; uncorrected) as well as beta increase (*p* < 0.05; uncorrected). After multiple comparison corrections for findings that did not have a specific *a priori* hypothesis, only the beta increase in the stimulated negative condition at 10 Hz remained significant (*p* < 0.05).

### Comparison of scalp EEG power features

**Figure 3:**
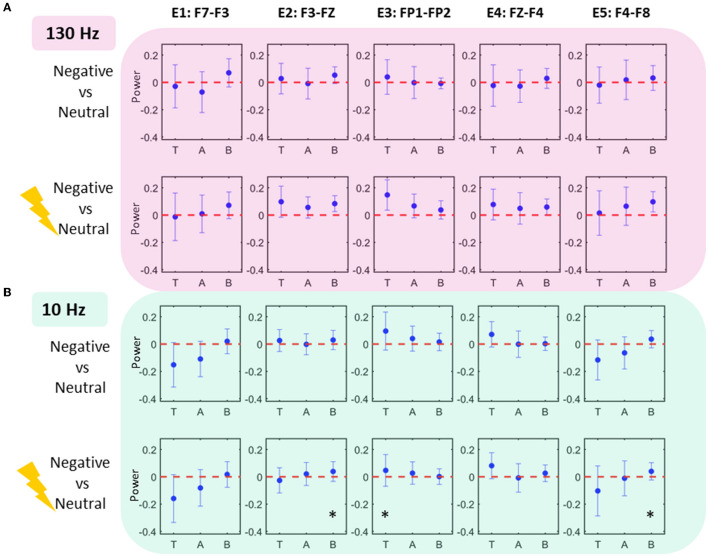
Scalp EEG power modulations. Each column shows the scalp EEG electrode power modulations in 10 Hz condition **(A)** and 130 Hz condition **(B)**. Effects (significance uncorrected) are seen in 10 Hz task at electrodes E2, E3, and E5 showing an increase in theta and beta power.

Figure 3 shows comparisons of scalp EEG LPR features extracted from the conditions in two tasks. Comparisons were statistically tested for effects only at theta, alpha, and beta bands (where significance was already established for LFP data) at five bipolar electrodes. All the effects were found in LPR comparison of stimulated negative condition to neutral in 10 Hz stimulation task: beta increases were found at E_2_ and E_5_ (*p* < 0.05; uncorrected), whereas a theta increase was located at E_3_ (*p* < 0.05; uncorrected).

### Power interaction across frequency bands and STN contacts

**Figure 4:**
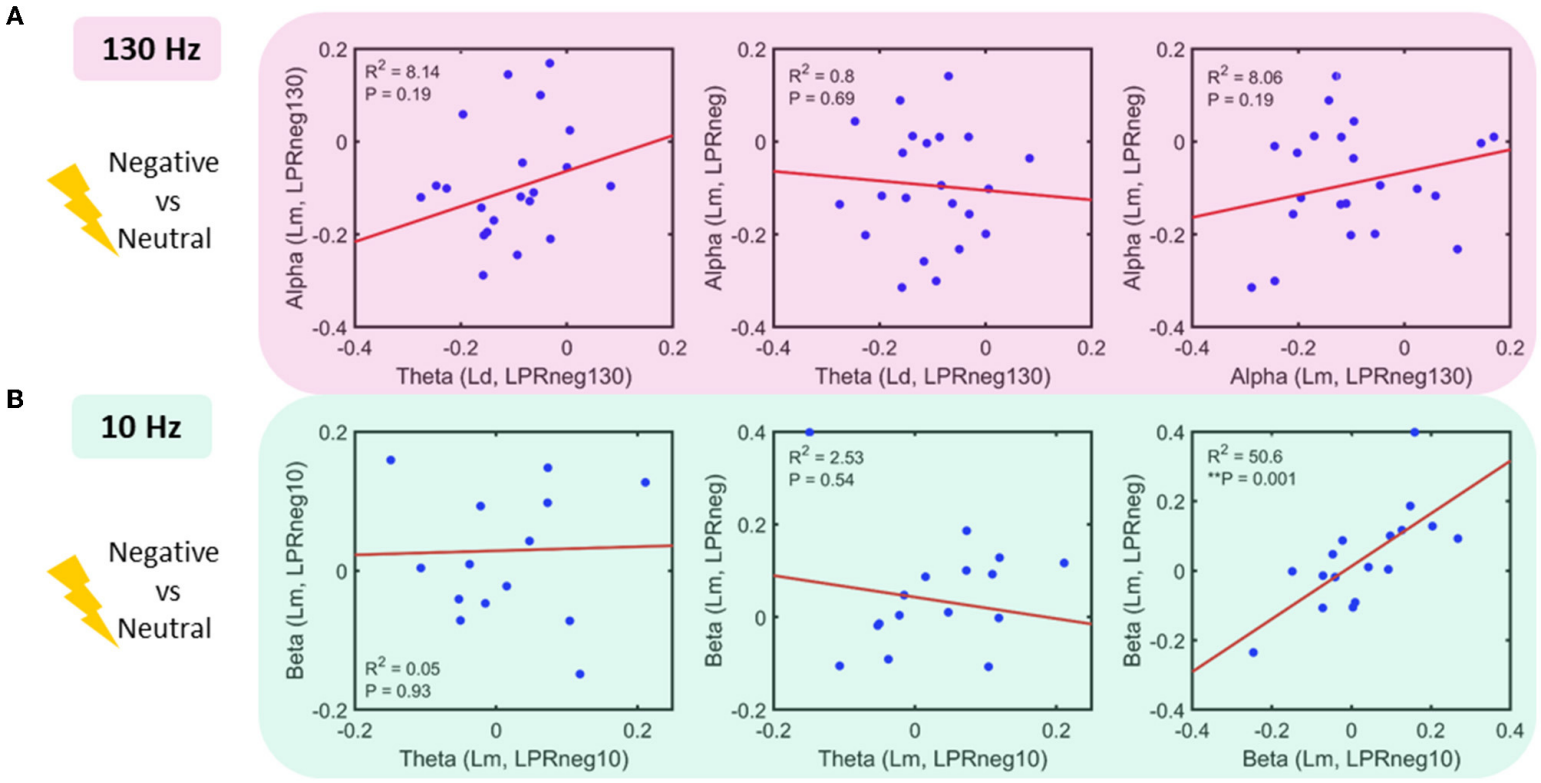
Power interactions between frequency bands **(A)** 130 Hz stim task: linear regression fit in pairs of the band as a function of location and power (log of power ratio–LPR, see methods): No significant relationship was found **(B)** 10 Hz stim task: linear regression fit in the pairs of a band as a function of location and power. Beta power for 10 Hz stimulation and unstimulated condition shows a significant positive relationship.

Following the results represented in Figure 2, a regression analysis was conducted across pairs of bands, each as a function of locations and negative affect conditions (Figure 4). For the 130 Hz stim task, the following relationships were sought: *theta* (Lr, LPR_neg130_) vs. *alpha* (Lm, LPR_neg130_), *theta* (Ld, LPR_neg130_) vs. *alpha* (Lm, LPR_neg_), and *alpha* (Lm, LPR_neg130_) vs. *alpha* (Lm, LPR_neg_). Only *beta* (Lm, LPR_neg10_) vs. *beta* (Lm, LPR_neg_) for 10 Hz stimulation tasks showed a significant relationship even after multiple comparison corrections. For the 10 Hz stim task, the following relationships were sought: *theta* (Lm, LPR_neg10_) vs. *beta* (Lm, LPR_neg10_), *theta* (Lm, LPR_neg10_) vs. *beta* (Lm, LPR_neg_), and *beta* (Lm, LPR_neg10_) vs. *beta* (Lm, LPR_neg_).

## Discussion

Our findings highlight the role of acute one-second right STN stimulation at 10 and 130 Hz on power modulations of contralateral STN LFP recordings during the subjective evaluation of negative imagery. We previously reported the effects of stimulation on subjective valence showing a shift toward positive subjective valence with 10 Hz stimulation but not 130 Hz stimulation (Mandali et al., 2021) and, in this study, investigated the physiological effects.

As hypothesized, in the 130 Hz stimulation condition, we show a decrease in alpha power to negative vs. neutral images irrespective of stimulation. This is consistent with previous findings in the literature of STN LFP recordings of a decrease in alpha power, or desynchronization, to affectively valenced images without stimulation (Huebl et al., 2014; Schubring and Schupp, 2019). This is also consistent with our demonstration of the lack of effect of acute high-frequency stimulation at 130 Hz on subjective valence as we previously reported (Mandali et al., 2021). We further show that acute alpha-specific frequency stimulation presumably was associated with a loss of this expected decrease or desynchronization in alpha power to negative images suggesting the capacity to facilitate the synchronization of alpha and enhance power. Additionally, we also observed enhanced synchronization in the frequency adjacent to that being stimulated with enhanced *beta* power, bands above that being stimulated, but not in more distant frequencies such as *delta* or *gamma*. This effect was also observed in a trend increase in prefrontal scalp EEG theta and beta bands. This physiological generalization may be related to the nature of our stimulation which was time-locked to a cognitive task but not phase-locked to the individual's specific frequency which may be associated with greater specificity as shown for treating tremors in PD (Little et al., 2013; Cagnan et al., 2017).

We further show a significant correlation in beta power to negative images across both the stimulated and unstimulated conditions along with a trend increase (p uncorrected) in beta power to negative images without stimulation within the alpha stimulation condition. These findings were not observed in neutral images or within the 130 Hz stimulation condition. This highlights a potential cognitive generalization effect that might reflect a generalization of a learned association between stimulation and negative valenced imagery. Acute alpha stimulation of negative imagery appears to enhance adjacent beta power to not only stimulated negative imagery but also other unstimulated negative imagery but critically and not to neutral stimuli. This suggests a potential learned association between negative imagery and 10 Hz stimulation on a physiological measure that then generalizes to imagery with similar negatively valenced features but not to neutral valenced features.

The duration of stimulation may lead to varied stimulation effects. For instance, local firing rates of neurons have been shown to increase after short bursts of high-frequency STN stimulation but are suppressed with prolonged stimulation (Lee et al., 2009). These observations may be more specific to high-frequency stimulation. In this study, we contrasted acute stimulation of similar short 1-s duration with differing frequencies and suggest our findings are frequency specific.

The role of the alpha ERD in subjective valence requires further investigations, and in this study, it was not specifically addressed. Alpha activity is most prevalent during resting wakefulness with closed eyes and decreases with enhanced attentional states. Modulation of alpha power with neurofeedback has been shown to modulate attention (Bagherzadeh et al., 2020; Deiber et al., 2020). Decreasing attention specifically toward negative imagery might also decrease the cognitive evaluation of subjective valence.

Current stimulation protocols for PD use high-frequency clinical stimulation of 130 or 160 Hz. Although this leads to dramatic reductions of motor symptoms, affective symptoms may linger (Campbell et al., 2012). Our results open up various avenues to tackle these mood symptoms. For instance, acute stimulation protocols could be paired up with neurofeedback training to modify emotional dysfunction not just in PD but other affective disorders amenable to DBS such as depression and obsessive-compulsive disorder. For example, DBS of habenula has shown promise for treating depression and alpha signatures to be correlated with depression severity (Sonkusare et al., 2022a) or dynamics of amygdala-prefrontal cortex circuitry in emotion processing (Sonkusare et al., 2022b) which can be targeted for neurofeedback training.

This study has some limitations. We stimulated the right STN but recorded it from the left STN as we were unable to stimulate and record from the same contacts. We did not analyze physiological data during the stimulation itself or 0.2 seconds immediately after stimulation end to avoid stimulation artifact and rebound effects although it remains plausible that our findings may still be contaminated by rebound or withdrawal effects rather than a specific role of stimulation influencing synchronization. Although newer methods for stimulation artifacts removal have been developed (Dastin-van Rijn et al., 2010), it is not a trivial issue. Future studies can build on our study to implement and validate artifact removal techniques. Additionally, the data were acquired 1 day after surgery, and “stun effects” may linger longer than this (Mestre et al., 2016). However, most past findings suggest changes in both impedance and LFPs occur after electrode implantation, particularly within the first 24 h (Williams et al., 2007; Lempka et al., 2010; Rosa et al., 2010). Furthermore, the logistical constraints and surgical setting of the data acquisition in the current study precluded longer waiting times to acquire the data. Finally, left/right asymmetry of the STN function has been an issue of ongoing investigations with some studies suggesting a lack of such differentiation (Kühn et al., 2005b; Buot and Yelnik, 2012; Lambert et al., 2012). However, future studies with a greater sample size are needed to clarify if stimulation of left asymmetry and recording from right asymmetry and vice-versa has a differential pattern of affecting the emotional behavior and spectral profile of STN signals. STN stimulation and its effects on scalp EEG, especially the high-density EEG are warranted which can delineate the left/right asymmetry effects of STN stimulation.

In conclusion, we show the capacity for acute low-frequency STN stimulation to synchronize physiological activity at the same frequency with evidence for physiological and cognitive generalization effects. Together, this provides evidence for the role of precision neuromodulation approaches and closed-loop deep brain stimulation for the advancement of neurological and neuropsychiatric therapies.

## Data availability statement

The data for this project were acquired from patients undergoing clinical care and consenting for additional research protocols. Researchers wishing to access these data will require local ethics approval and a data sharing agreement with Ruijin Hospital, Shanghai, China. Further inquiries can be directed to the corresponding author.

## Ethics statement

The studies involving human participants were reviewed and approved by Ethics Committee of Ruijin Hospital, Shanghai Jiao Tong University School of Medicine, Shanghai, China. The patients/participants provided their written informed consent to participate in this study.

## Author contributions

SS: formal analysis, investigation, methodology, resources, software, visualization, writing—original draft, review, and editing. NM: data curation, formal analysis, investigation, methodology, resources, software, visualization, and writing—review and editing. AM: conceptualization, data acquisition, data curation, resources, and writing—review and editing. QD: data acquisition, data curation, formal analysis, software, and visualization. LW: data acquisition and data curation. YZ: data acquisition, data curation, funding acquisition, investigation, and project administration. BS: data acquisition, funding acquisition, and project administration. DL: experimental planning and logistics, funding of the experimental study, patient recruitment, surgical evaluation, and data acquisition. VV: conceptualization, funding acquisition, project administration, methodology, supervision, and writing—review and editing. All authors contributed to the article and approved the submitted version.
