# Supplementary material for: Time-locked acute alpha-frequency stimulation of subthalamic nuclei during the evaluation of emotional stimuli and its effect on power modulation
Source: Front Hum Neurosci. 2023 Jul 27;17:1181635. doi: 10.3389/fnhum.2023.1181635 (PMC10415014; doi:10.3389/fnhum.2023.1181635)
Supplement: Supplementary file 1 [file Table_1.DOCX]

**Time-locked acute alpha-frequency stimulation of subthalamic nuclei during evaluation of emotional stimuli and its effect on power modulation**

Naeem Muhammad^1†^, Saurabh Sonkusare^1†^, Ding Qiong^2^, Linbin Wang^2^, Alekhya Mandali^1^, Yi Jie Zhao^3^, Bomin Sun^2^, Valerie Voon*^1, 2, 3^

**Supplementary Table 1: Subjects in each condition and the corresponding MNI contacts**

| **Subj id** |  | **No Stim**  **MNI coordinates** | **10 Hz Stim**  **MNI coordinates** | **130 Hz Stim**  **MNI coordinates** |
| --- | --- | --- | --- | --- |
|  | Contact | X Y Z | X Y Z | X Y Z |
| 21 | R0  R1  R2  R3  L0  L1  L2  L3 |  |  | 12.1 -15.4 -8.6  13.4 -14.1 -5.5  14.8 -12.9 -2.4  16.1 -11.7 0.7  -10.3 -16.8 -7.9  -11.4 -14.9 -5.0  -12.5 -13.0 -2.0  -13.7 -11.1 0.9 |
| 22 | R0  R1  R2  R3  L0  L1  L2  L3 |  | 11.6 -17.0 -9.1  12.8 -15.2 -6.7  14.0 -13.5 -3.7  15.2 -11.7 -0.8  -9.8 -17.3 -7.5  -10.8 -15.7 -4.5  -11.9 -14.1 -1.5  -12.9 -12.5 1.5 | 11.6 -17.0 -9.1  12.8 -15.2 -6.7  14.0 -13.5 -3.7  15.2 -11.7 -0.8  -9.8 -17.3 -7.5  -10.8 -15.7 -4.5  -11.9 -14.1 -1.5  -12.9 -12.5 1.5 |
| 23 | R0  R1  R2  R3  L0  L1  L2  L3 |  | 11.6 -14.1 -9.8  12.8 -13.0 -6.4  14.0 -11.9 -3.0  15.1 -10.8 0.4  -12.8 -15.3 -7.5  -14.0 -14.1 -4.3  -15.2 -12.8 -1.2  -16.3 -11.6 2.0 | 11.6 -14.1 -9.8  12.8 -13.0 -6.4  14.0 -11.9 -3.0  15.1 -10.8 0.4  -12.8 -15.3 -7.5  -14.0 -14.1 -4.3  -15.2 -12.8 -1.2  -16.3 -11.6 2.0 |
| 25 | R0  R1  R2  R3  L0  L1  L2  L3 | 10.6 -14.4 -8.6  11.2 -12.9 -5.3  11.9 -11.4 -2.0  12.5 -9.9 1.3  -10.8 -15.9 -9.4  -12.2 -15.0 -6.2  -13.5 -14.0 -3.0  -14.9 -13.1 0.2 | 10.6 -14.4 -8.6  11.2 -12.9 -5.3  11.9 -11.4 -2.0  12.5 -9.9 1.3  -10.8 -15.9 -9.4  -12.2 -15.0 -6.2  -13.5 -14.0 -3.0  -14.9 -13.1 0.2 | 10.6 -14.4 -8.6  11.2 -12.9 -5.3  11.9 -11.4 -2.0  12.5 -9.9 1.3  -10.8 -15.9 -9.4  -12.2 -15.0 -6.2  -13.5 -14.0 -3.0  -14.9 -13.1 0.2 |
| 26 | R0  R1  R2  R3  L0  L1  L2  L3 | 10.0 -16.7 -10.0  10.9 -15.2 -7.0  11.7 -13.8 -4.0  12.6 -12.3 -1.0  -11.0 -18.6 -9.4  -12.2 -17.2 -6.7  -13.4 -15.8 -4.0  -14.6 -14.3 -1.2 | 10.0 -16.7 -10.0  10.9 -15.2 -7.0  11.7 -13.8 -4.0  12.6 -12.3 -1.0  -11.0 -18.6 -9.4  -12.2 -17.2 -6.7  -13.4 -15.8 -4.0  -14.6 -14.3 -1.2 | 10.0 -16.7 -10.0  10.9 -15.2 -7.0  11.7 -13.8 -4.0  12.6 -12.3 -1.0  -11.0 -18.6 -9.4  -12.2 -17.2 -6.7  -13.4 -15.8 -4.0  -14.6 -14.3 -1.2 |
| 27 | R0  R1  R2  R3  L0  L1  L2  L3 | 13.4 -14.3 -8.6  14.8 -12.9 -5.5  16.1 -11.5 -2.3  17.5 -10.0 0.9  -12.9 -16.7 -6.1  -13.7 -15.2 -2.9  -14.5 -13.8 0.4  -15.3 -12.4 3.7 |  | 13.4 -14.3 -8.6  14.8 -12.9 -5.5  16.1 -11.5 -2.3  17.5 -10.0 0.9  -12.9 -16.7 -6.1  -13.7 -15.2 -2.9  -14.5 -13.8 0.4  -15.3 -12.4 3.7 |
| 29 | R0  R1  R2  R3  L0  L1  L2  L3 | 13.8 -16.3 -7.4  14.9 -14.7 -4.5  16.0 -13.1 -1.5  17.0 -11.6 1.5  -13.4 -17.7 -8.7  -14.3 -16.4 -5.4  -15.1 -15.1 -2.2  -16.0 -13.7 1.0 | 13.8 -16.3 -7.4  14.9 -14.7 -4.5  16.0 -13.1 -1.5  17.0 -11.6 1.5  -13.4 -17.7 -8.7  -14.3 -16.4 -5.4  -15.1 -15.1 -2.2  -16.0 -13.7 1.0 | 13.8 -16.3 -7.4  14.9 -14.7 -4.5  16.0 -13.1 -1.5  17.0 -11.6 1.5  -13.4 -17.7 -8.7  -14.3 -16.4 -5.4  -15.1 -15.1 -2.2  -16.0 -13.7 1.0 |
| 30 | R0  R1  R2  R3  L0  L1  L2  L3 | 10.7 -15.1 -10.4  11.8 -13.8 -7.2  13.0 -12.4 -4.0  14.1 -11.1 -0.8  -11.4 -15.7 -10.7  -12.6 -14.0 -7.9  -13.8 -12.3 -5.1  -14.9 -10.5 -2.3 | 10.7 -15.1 -10.4  11.8 -13.8 -7.2  13.0 -12.4 -4.0  14.1 -11.1 -0.8  -11.4 -15.7 -10.7  -12.6 -14.0 -7.9  -13.8 -12.3 -5.1  -14.9 -10.5 -2.3 | 10.7 -15.1 -10.4  11.8 -13.8 -7.2  13.0 -12.4 -4.0  14.1 -11.1 -0.8  -11.4 -15.7 -10.7  -12.6 -14.0 -7.9  -13.8 -12.3 -5.1  -14.9 -10.5 -2.3 |
| 33 | R0  R1  R2  R3  L0  L1  L2  L3 |  | 10.8 -15.0 -9.0  11.8 -14.1 -5.8  12.8 -13.1 -2.5  3.9 -12.2 0.7  10.3 -15.8 -8.7  -11.3 -14.9 -5.4  -12.4 -14.0 -2.1  -13.4 -13.0 1.1 | 10.8 -15.0 -9.0  11.8 -14.1 -5.8  12.8 -13.1 -2.5  3.9 -12.2 0.7  10.3 -15.8 -8.7  -11.3 -14.9 -5.4  -12.4 -14.0 -2.1  -13.4 -13.0 1.1 |
| 34 | R0  R1  R2  R3  L0  L1  L2  L3 | 12.7 -14.3 -8.7  13.9 -12.9 -5.8  15.1 -11.5 -3.0  16.3 -10.2 -0.2  -10.4 -15.3 -7.0  -11.4 -13.8 -4.0  -12.3 -12.3 -0.9  -13.3 -10.8 2.1 |  | 12.7 -14.3 -8.7  13.9 -12.9 -5.8  15.1 -11.5 -3.0  16.3 -10.2 -0.2  -10.4 -15.3 -7.0  -11.4 -13.8 -4.0  -12.3 -12.3 -0.9  -13.3 -10.8 2.1 |
| 38 | R0  R1  R2  R3  L0  L1  L2  L3 | 11.1 -17.6 -8.4  12.1 -16.0 -5.4  13.2 -14.5 -2.3  14.2 -13.0 0.7  -10.2 -17.4 -7.7  -11.0 -16.4 -4.4  -11.8 -15.3 -1.0  -12.6 -14.3 2.3 |  | 11.1 -17.6 -8.4  12.1 -16.0 -5.4  13.2 -14.5 -2.3  14.2 -13.0 0.7  -10.2 -17.4 -7.7  -11.0 -16.4 -4.4  -11.8 -15.3 -1.0  -12.6 -14.3 2.3 |
| 40 | R0  R1  R2  R3  L0  L1  L2  L3 | 12.1 -16.0 -8.3  13.2 -14.6 -5.1  14.3 -13.1 -1.9  15.3 -11.7 1.3  -11.5 -16.7 -7.4  -12.2 -15.3 -4.3  -12.9 -13.8 -1.2  -13.6 -12.3 2.0 | 12.1 -16.0 -8.3  13.2 -14.6 -5.1  14.3 -13.1 -1.9  15.3 -11.7 1.3  -11.5 -16.7 -7.4  -12.2 -15.3 -4.3  -12.9 -13.8 -1.2  -13.6 -12.3 2.0 | 12.1 -16.0 -8.3  13.2 -14.6 -5.1  14.3 -13.1 -1.9  15.3 -11.7 1.3  -11.5 -16.7 -7.4  -12.2 -15.3 -4.3  -12.9 -13.8 -1.2  -13.6 -12.3 2.0 |
| 43 | R0  R1  R2  R3  L0  L1  L2  L3 | 10.8 -16.3 -10.2  12.0 -14.7 -7.2  3.2 -13.1 -4.2  14.4 -11.5 -1.2  -11.6 -19.5 -7.9  -12.3 -17.6 -5.0  -13.0 -15.7 -2.1  -13.8 -13.9 0.8 |  | 10.8 -16.3 -10.2  12.0 -14.7 -7.2  3.2 -13.1 -4.2  14.4 -11.5 -1.2  -11.6 -19.5 -7.9  -12.3 -17.6 -5.0  -13.0 -15.7 -2.1  -13.8 -13.9 0.8 |
| 44 | R0  R1  R2  R3  L0  L1  L2  L3 | 10.8 -15.0 -10.3  11.9 -13.9 -7.1  13.0 -12.7 -3.9  14.0 -11.6 -0.7  -11.3 -16.1 -9.2  -12.4 -14.8 -6.3  -13.4 -13.4 -3.5  -14.4 -12.0 -0.6 | 10.8 -15.0 -10.3  11.9 -13.9 -7.1  13.0 -12.7 -3.9  14.0 -11.6 -0.7  -11.3 -16.1 -9.2  -12.4 -14.8 -6.3  -13.4 -13.4 -3.5  -14.4 -12.0 -0.6 |  |
| 45 | R0  R1  R2  R3  L0  L1  L2  L3 | 11.2 -17.5 -10.7  12.2 -16.1 -7.7  13.3 -14.7 -4.8  14.4 -13.3 -1.9  -11.8 -15.8 -9.7  -13.1 -14.8 -6.7  -14.3 -13.8 -3.7  -15.6 -12.7 -0.7 |  | 11.2 -17.5 -10.7  12.2 -16.1 -7.7  13.3 -14.7 -4.8  14.4 -13.3 -1.9  -11.8 -15.8 -9.7  -13.1 -14.8 -6.7  -14.3 -13.8 -3.7  -15.6 -12.7 -0.7 |
| 46 | R0  R1  R2  R3  L0  L1  L2  L3 | 11.4 -16.3 -9.4  12.6 -14.8 -6.0  13.8 -13.2 -2.6  15.0 -11.7 0.9  -10.7 -16.9 -7.5  -11.7 -15.3 -4.1  -12.7 -13.7 -0.7  -13.6 -12.0 2.8 |  | 11.4 -16.3 -9.4  12.6 -14.8 -6.0  13.8 -13.2 -2.6  15.0 -11.7 0.9  -10.7 -16.9 -7.5  -11.7 -15.3 -4.1  -12.7 -13.7 -0.7  -13.6 -12.0 2.8 |
| 50 | R0  R1  R2  R3  L0  L1  L2  L3 | 12.1 -14.8 -8.8  13.1 -14.0 -5.2  14.1 -13.2 -1.5  15.1 -12.4 2.1  -12.4 -15.4 -10.3  -13.5 -14.6 -6.7  -14.6 -13.8 -3.0  -15.7 -12.9 0.6 | 12.1 -14.8 -8.8  13.1 -14.0 -5.2  14.1 -13.2 -1.5  15.1 -12.4 2.1  -12.4 -15.4 -10.3  -13.5 -14.6 -6.7  -14.6 -13.8 -3.0  -15.7 -12.9 0.6 | 12.1 -14.8 -8.8  13.1 -14.0 -5.2  14.1 -13.2 -1.5  15.1 -12.4 2.1  -12.4 -15.4 -10.3  -13.5 -14.6 -6.7  -14.6 -13.8 -3.0  -15.7 -12.9 0.6 |
| 52 | R0  R1  R2  R3  L0  L1  L2  L3 | 13.2 -16.1 -8.8  13.8 -15.3 -6.0  14.9 -14.0 -3.0  16.4 -12.3 -0.2  -10.6 -18.4 -8.4  -11.3 -16.7 -5.7  -12.1 -15.1 -3.0  -12.9 -13.4 -0.3 | 13.2 -16.1 -8.8  13.8 -15.3 -6.0  14.9 -14.0 -3.0  16.4 -12.3 -0.2  -10.6 -18.4 -8.4  -11.3 -16.7 -5.7  -12.1 -15.1 -3.0  -12.9 -13.4 -0.3 | 13.2 -16.1 -8.8  13.8 -15.3 -6.0  14.9 -14.0 -3.0  16.4 -12.3 -0.2  -10.6 -18.4 -8.4  -11.3 -16.7 -5.7  -12.1 -15.1 -3.0  -12.9 -13.4 -0.3 |
| 54 | R0  R1  R2  R3  L0  L1  L2  L3 | 11.4 -17.6 -11.9  12.7 -16.4 -8.5  14.0 -15.2 -5.1  15.2 -14.0 -1.7  -11.2 -19.2 -9.2  -12.1 -17.8 -5.8  -12.9 -16.4 -2.4  -13.8 -15.0 0.9 | 11.4 -17.6 -11.9  12.7 -16.4 -8.5  14.0 -15.2 -5.1  15.2 -14.0 -1.7  -11.2 -19.2 -9.2  -12.1 -17.8 -5.8  -12.9 -16.4 -2.4  -13.8 -15.0 0.9 | 11.4 -17.6 -11.9  12.7 -16.4 -8.5  14.0 -15.2 -5.1  15.2 -14.0 -1.7  -11.2 -19.2 -9.2  -12.1 -17.8 -5.8  -12.9 -16.4 -2.4  -13.8 -15.0 0.9 |
| 62 | R0  R1  R2  R3  L0  L1  L2  L3 |  | 10.6 -13.8 -10.6  10.4 -13.8 -7.5  10.9 -12.3 -4.7  13.4 -8.6 -2.4  -8.2 -14.3 -9.2  -8.7 -12.9 -6.2  -9.2 -11.5 -3.3  -9.7 -10.1 -0.4 | 10.6 -13.8 -10.6  10.4 -13.8 -7.5  10.9 -12.3 -4.7  13.4 -8.6 -2.4  -8.2 -14.3 -9.2  -8.7 -12.9 -6.2  -9.2 -11.5 -3.3  -9.7 -10.1 -0.4 |
| 67 | R0  R1  R2  R3  L0  L1  L2  L3 |  | 11.9 -15.9 -7.4  12.8 -14.0 -4.6  13.7 -12.1 -1.8  14.5 -10.3 1.1  12.7 -15.6 -8.3  -13.6 -14.0 -5.4  -14.5 -12.3 -2.4  -15.4 -10.7 0.5 | 11.9 -15.9 -7.4  12.8 -14.0 -4.6  13.7 -12.1 -1.8  14.5 -10.3 1.1  12.7 -15.6 -8.3  -13.6 -14.0 -5.4  -14.5 -12.3 -2.4  -15.4 -10.7 0.5 |
| 68 | R0  R1  R2  R3  L0  L1  L2  L3 |  | 11.1 -15.3 -7.5  12.3 -14.1 -4.1  13.5 -12.9 -0.8  14.7 -11.7 2.5  -11.4 -15.6 -6.3  -12.4 -14.5 -3.0  -13.5 -13.4 0.3  -14.6 -12.3 3.7 | 11.1 -15.3 -7.5  12.3 -14.1 -4.1  13.5 -12.9 -0.8  14.7 -11.7 2.5  -11.4 -15.6 -6.3  -12.4 -14.5 -3.0  -13.5 -13.4 0.3  -14.6 -12.3 3.7 |
| 69 | R0  R1  R2  R3  L0  L1  L2  L3 |  | 9.7 -16.1 -10.3  11.0 -15.0 -7.0  12.3 -14.0 -3.7  13.6 -12.9 -0.4  -13.4 -14.2 -6.3  -14.4 -12.7 -3.1  -15.3 -11.2 0.1  -16.3 -9.7 3.2 | 9.7 -16.1 -10.3  11.0 -15.0 -7.0  12.3 -14.0 -3.7  13.6 -12.9 -0.4  -13.4 -14.2 -6.3  -14.4 -12.7 -3.1  -15.3 -11.2 0.1  -16.3 -9.7 3.2 |
| 70 | R0  R1  R2  R3  L0  L1  L2  L3 |  | 12.2 -16.0 -8.8  13.4 -14.5 -5.7  13.4 -14.5 -5.7  15.8 -11.7 0.5  -11.4 -15.3 -7.3  -12.5 -14.2 -3.9  -13.7 -13.1 -0.5  -14.8 -12.1 2.9 | 12.2 -16.0 -8.8  13.4 -14.5 -5.7  13.4 -14.5 -5.7  15.8 -11.7 0.5  -11.4 -15.3 -7.3  -12.5 -14.2 -3.9  -13.7 -13.1 -0.5  -14.8 -12.1 2.9 |
